# CD4 Depletion or CD40L Blockade Results in Antigen-Specific Tolerance in a Red Blood Cell Alloimmunization Model

**DOI:** 10.3389/fimmu.2017.00907

**Published:** 2017-08-07

**Authors:** Prabitha Natarajan, Dong Liu, Seema R. Patel, Manjula Santhanakrishnan, Daniel Beitler, Jingchun Liu, David R. Gibb, Justine S. Liepkalns, David J. Madrid, Stephanie C. Eisenbarth, Sean R. Stowell, Jeanne E. Hendrickson

**Affiliations:** ^1^Department of Laboratory Medicine, Yale University School of Medicine, New Haven, CT, United States; ^2^Department of Pathology and Laboratory Medicine, Emory University School of Medicine, Atlanta, GA, United States; ^3^Department of Pediatrics, Yale University School of Medicine, New Haven, CT, United States; ^4^Department of Immunobiology, Yale University School of Medicine, New Haven, CT, United States

**Keywords:** red blood cell, alloimmunization, tolerance, CD40L blockade, T-cells

## Abstract

Approximately 3–10% of human red blood cell (RBC) transfusion recipients form alloantibodies to non-self, non-ABO blood group antigens expressed on donor RBCs, with these alloantibodies having the potential to be clinically significant in transfusion and pregnancy settings. However, the majority of transfused individuals never form detectable alloantibodies. Expanding upon observations that children initially transfused with RBCs at a young age are less likely to form alloantibodies throughout their lives, we hypothesized that “non-responders” may not only be ignorant of antigens on RBCs but instead tolerized. We investigated this question in a reductionist murine model, in which transgenic donors express the human glycophorin A (hGPA) antigen in an RBC-specific manner. Although wild-type mice treated with poly IC and transfused with hGPA RBCs generated robust anti-hGPA IgG alloantibodies that led to rapid clearance of incompatible RBCs, those transfused in the absence of an adjuvant failed to become alloimmunized. Animals depleted of CD4^+^ cells or treated with CD40L blockade prior to initial hGPA RBC exposure, in the presence of poly IC, failed to generate detectable anti-hGPA IgG alloantibodies. These non-responders to a primary transfusion remained unable to generate anti-hGPA IgG alloantibodies upon secondary hGPA exposure and did not prematurely clear transfused hGPA RBCs even after their CD4 cells had returned or their CD40L blockade had resolved. This observed tolerance was antigen (hGPA) specific, as robust IgG responses to transfused RBCs expressing a third-party antigen occurred in all studied groups. Experiments completed in an RBC alloimmunization model that allowed evaluation of antigen-specific CD4^+^ T-cells (HOD (hen egg lysozyme, ovalbumin, and human duffy^b^)) demonstrated that CD40L blockade prevented the expansion of ovalbumin 323-339 specific T-cells after HOD RBC transfusion and also prevented germinal center formation. Taken together, our data suggest that recipients may indeed become tolerized to antigens expressed on RBCs, with the recipient’s immune status upon initial RBC exposure dictating future responses. Although questions surrounding mechanism(s) and sustainability of tolerance remain, these data lay the groundwork for future work investigating RBC immunity versus tolerance in reductionist models and in humans.

## Introduction

Transfusion of red blood cells (RBCs) is the most common procedure completed during hospitalizations (1). Although RBC transfusions are often lifesaving, they are not without risk. In addition to complications such as febrile reactions, bacterial contamination, or infectious disease transmission (2, 3), there is a risk of formation of antibodies to non-self blood group antigens (RBC alloimmunization). Although transfusions given in non-emergent situations are ABO and RhD compatible between donor and recipient, they are typically not matched for other blood group antigens such as C/c, E/e, K/k, Jka/Jkb, Fya/Fyb, S/s, etc. Overall there are more than 30 blood groups and hundreds of variants of these groups (4, 5).

Red blood cell alloimmunization rates vary depending on patient population studied and antibody detection techniques. As few as 1% of transfused oncology patients treated with chemotherapy have been described to be alloimmunized, with up to 40–50% of transfused patients with sickle-cell disease being alloimmunized (6). Because each transfusion exposes a recipient to many non-self blood group antigens, the variation in alloimmunization rates between different patient groups is not fully understood. Despite this exposure to non-self antigens, the majority of transfused individuals never develop RBC alloantibodies. Factors such as the recipient inflammatory status at the time of RBC exposure (7) and underlying autoimmunity (8) are thought to be important variables in the predisposition to develop RBC alloantibodies.

The clinical significance of RBC alloantibodies in transfusion, pregnancy, and transplantation settings cannot be overstated. These antibodies may lead to hemolytic transfusion reactions, which are one of the leading causes of transfusion-associated death reported yearly to the FDA (9). Alloantibodies in those requiring transfusion make locating compatible RBC units difficult and, at times, impossible (10). They contribute to bystander hemolysis in some patients with sickle-cell disease, which can be fatal (11). In a pregnancy setting, RBC alloantibodies may result in hemolytic disease of the newborn (HDFN) (12, 13). In a hematopoietic stem cell transplantation setting, these antibodies may impact processing of the graft or engraftment (14), and in a solid organ transplant setting, RBC antibodies against antigens expressed on cells in the kidney (e.g., those in the Kidd family) have been implicated in graft rejection (15, 16).

Strategies to prevent RBC alloantibody development are currently limited, with our incomplete understanding of recipient immunologic responses to RBC transfusion therapy playing a contributing role. The most effective strategy includes transfusion avoidance, which is not feasible in many situations. Another strategy employed includes providing RBC units from donors matching recipients at high risk for alloimmunization at antigens beyond ABO and RhD (“phenotypic matching”) (17). However, this strategy is not entirely effective due to the inability to provide RBCs matched at all antigen sites, due to Rh variant alleles in donors/recipients that may not be appreciated by phenotyping alone (18, 19) and due to patients being transfused at multiple different locations including those that may not provide such matched RBCs (20). At the present time, there are no known immunologic or genetic “signatures” of patients at high or low risk of becoming alloimmunized, although the identification of such a signature would be quite valuable (21).

We study murine models of RBC alloimmunization in our laboratory to better understand the immunologic steps resulting in alloantibody formation and to investigate rationale strategies to mitigate the formation of such antibodies (22). Our models allow reductionist studies to be completed that are simply not feasible in humans, including the study of controlled blood group antigenic differences between donor and recipients. Although the ability of a human recipient to respond to a particular antigen on a transfused RBC may depend on the HLA type of the recipient (23), our murine transfusion recipients are MHC identical and thus all capable of presenting the blood group antigens being studied. Further, our models allow for the comparison of single variable changes of the recipient’s immune system on alloantibody induction.

In this current study, we investigate the steps leading to an alloimmune response in a model in which murine donor RBCs express the human glycophorin A (hGPA) blood group antigen (24). Others and we have previously observed that transfused hGPA RBCs do not result in an anti-hGPA RBC alloantibody response unless the recipients are treated with an adjuvant around the time of the transfusion or unless the transfused RBCs are co-infused with an adjuvant (25, 26). Polyinosinic-polycytidylic acid, a mimetic of viral double-stranded RNA, is one such adjuvant known to enhance RBC alloimmunization in multiple murine models (27). These past data led to the hypothesis that the hGPA RBC alloimmune response was CD4^+^ T-cell dependent and to the thought that co-stimulatory blockade might prevent alloimmunization altogether. Herein, we describe conditions under which immunity versus tolerance can be induced by transfused hGPA RBCs, and we begin to investigate the mechanism(s) involved in these responses.

## Materials and Methods

### Mice

FVB/NCr mice were purchased from Charles River Laboratories (Wilmington, MA, USA). C57BL/6 mice were purchased from the National Cancer Institute (Frederick, MD, USA) or Taconic (Hudson, NY, USA). Transgenic mice expressing hGPA (24) on the RBCs on FVB genetic background (H2-q) generously provided by the New York Blood Center and HOD mice that have RBC-specific expression of *h*en egg lysozyme, *o*valbumin, and human *D*uffy b (28) or the KEL glycoprotein (29) at a high density (“KEL^hi^”) were bred in a non-pathogen-free facility at Yale University. OTII mice on a CD45.1 C57BL/6 (H2b) background whose CD4^+^ T-cells have transgenic T-cell receptors specific for ovalbumin were also bred at Yale. Transfusion-recipient mice were 8–12 weeks of age and on FVB background unless specified otherwise. All procedures and protocols were approved by Emory and Yale University’s Institutional Animal Care and Use Committee.

### CD4 Depletion and CD40L Blockade

For CD4 depletion experiments, a previously optimized dosing schedule was utilized (30): mice were given two IP injections of 200 µg anti-mouse CD4 monoclonal antibody (clone: GK1.5, BioXcell, West Lebanon, NH, USA), saline, or an isotype-matched control 2 days apart. 48 h after the second injection, mice were transfused. On day 7 post-transfusion, another injection of 300 µg GK1.5 antibody was given.

For CD40L blocking experiments, another previously optimized dosing schedule was utilized (31): all mice were given IP injections of 250 µg anti-mouse CD40L(CD154) monoclonal antibody (clone: MR1, BioXcell, West Lebanon, NH, USA), saline, or an isotype-matched control every 2 days starting on the day of transfusion until 6 days after the transfusion. Additional MR1 was also given on days 9 and 12.

### Transfusion

Mice were transfused (IV injection) with 75 µL of fresh packed transgenic hGPA or HOD RBCs that had been collected in the anticoagulant preservative solution CPDA-1 (citrate phosphorus dextrose adenine, Jorgensen Labs, Henry Schein, Melville, NY, USA) and filter leukoreduced over a Pall (East Hills, NY, USA) syringe filter. Some recipients were IP injected with 100 µg poly IC, polyinosinic-polycytidylic acid (Invivogen, San Diego, CA, USA), approximately 3–4 h before transfusion. FVB recipient mice were used for hGPA experiments as the hGPA donors are on an FVB background, and C57BL/6 mice were used for HOD experiments as the HOD donors are on a C57BL/6 background.

### Fluorescent Labeling for RBC Clearance

After collection and leukoreduction, donor hGPA or wild-type FVB/NCr RBCs were labeled with chloromethylbenzamido 1,1′-dioctadecyl-3,3,3′,3′-tetramethylindocarbocyanine perchlorate or 3,3′-dihexadecyloxacarbocyanine perchlorate according to the manufacturer’s instructions (Molecular Probes, Eugene, OR, USA) and as previously described (32). Recipient mice were transfused *via* IV tail vein with 75 µL of hGPA RBCs and a similar amount of wild-type RBCs. Survival of the transfused RBCs was determined by comparing the ratio of circulating hGPA RBCs to control RBCs in recipients longitudinally post-transfusion.

### Adoptive Transfer

Single splenic cell suspensions from 8 to 10 weeks old female donor CD45.1 OT-II mice were prepared using gentle mechanical disruption, followed by RBC lysis with AcK buffer (0.15 M NH4Cl, 10 mM KHCO3, and 0.1 mM EDTA). CD4^+^ T-cells were isolated using a mouse CD4^+^ T-cell-negative isolation selection kit (Stemcell Technologies, Vancouver, BC, Canada). Purified OTII CD4^+^ T-cells were retro-orbitally injected into recipient mice. Recipient C57BL/6 mice were transfused with HOD RBCs 24 h following adoptive transfer.

### Flow Cytometric Analysis

#### RBC Flow Cytometric Crossmatch

Levels of anti-hGPA/HOD alloantibodies in transfusion recipients were measured by a flow cytometric crossmatch assay as previously described (33) using IgM, total Igs, or IgG (BD Biosciences, San Jose, CA, USA). In brief, antigen-specific responses were determined by calculating an adjusted mean fluorescence intensity (MFI), which is the difference between the signal obtained with sera crossmatched with antigen-positive (hGPA/HOD) RBCs and that obtained with sera crossmatched with antigen-negative (FVB/NCr) RBCs. The adjusted MFI thus represents antibody (IgM, Igs, or IgG) that is specifically targeted against the non-self RBC antigen that the recipient was exposed to *via* transfusion. For the flow cytometric crossmatch assay, samples were analyzed on a four-color BD FACS Calibur or 8-color Miltenyi MACSQuant^®^ Analyzer with analysis completed using Flo Jo software.

#### Immune Cell Sub-Population Analysis

To determine frequencies and numbers of different cell populations, flow cytometry was performed on single-cell suspensions from bone marrow (BM) and spleen tissues longitudinally, at specified time points. In brief, spleens were harvested and homogenized into a single-cell suspension in Hank’s balanced salt solution (HBSS) using a 5-mL syringe plunger. Single cells from BM tissues were obtained by pipetting the tissue in and out several times in HBSS. For flow cytometric analysis of immune cells, RBCs were lysed using ammonium chloride and potassium bicarbonate salt solution. Cells were stained with different surface antibodies in buffer containing 0.1% EDTA and 0.01% bovine serum albumin. Immune cell subsets in splenocytes and BM cells were analyzed *via* flow cytometry using fluorochrome-conjugated monoclonal antibodies to mouse surface markers CD19 (clone#eBio1D3, eBiosciences), CD45R (B220, clone#RA3-6B2, eBiosciences), CD5 (clone#53-7.3, Biolegend), CD1d (clone#1B1, Biolenged), GL7 (Clone#GL7, Biolegend), CD95 (clone#Jo2, BD pharmingen), CD138 (Clone#281-2, Biolegend), TCRβ (Clone#H57-597, Biolegend), CD4 (Clone#GK1.5, Biolegend), CXCR5 (Clone#2G8, BD pharmingen), PD1 (Clone#29F.1A12, Biolegend), and BCL6 (Clone#K112-91, BD pharmingen) (BD Biosciences, San Jose, CA, USA or eBiosciences San Diego, CA, USA or BioLegend San Diego, CA, USA). To detect regulatory T-cells (Tregs), intracellular Foxp3 staining with fix and permeabilization kit was performed (eBiosciences, San Diego, CA, USA) according to the manufacturer’s instructions. Live cells were first gated using live/dead stain (BioLegend, San Diego, CA, USA). T-follicular helper (TFH) cells were defined as CD4^+^ CXCR5^hi^PD-1^+^ or CD4^+^BCL6^+^. Samples were analyzed on a LSR II flow cytometer (BD Biosciences, San Jose, CA, USA) or on Miltenyi MACSQuant^®^ Analyzer.

### Immunofluorescence

For analysis of germinal centers (GCs), spleens were cryopreserved in optimal cutting temperature medium, sectioned on a cryostat, and stained. Frozen spleens were sectioned at 8-µm thickness fixed in 4% paraformaldehyde (Electron Microscopy Sciences, Hatfield, PA, USA) washed in ice-cold PBS, and stained at room temperature for 60 min with anti-B220 (AF488), anti-CD4 (PE), and biotinylated PNA in 5% fetal calf serum/PBS solution. After primary staining, the slides were washed in ice-cold PBS three times and stained with streptavidin AF647. In some circumstances, AF647-conjugated GL7 was used for GC staining instead of PNA and observed similar staining patterns. For consistency, near middle sections of the spleens were used for staining and were then analyzed using an automated wide-field microscope (Nikon Eclipse Ti) and CCD camera (Qimaging Retiga 2000R) with NIS elements software. Number of GC was counted in the entire field of view of the spleen sections.

### Statistics

All statistical analyses were performed using Graph Pad Prism software (San Diego, CA, USA). The Mann–Whitney *U* test was used to determine significant differences between two groups, and the Kruskal–Wallis with Dunn’s post-test or a two-way ANOVA with Tukey’s multiple comparisons test were used when comparing more than two groups as indicated. Error bars represent one SD, and significance was determined by a *p*-value of <0.05.

## Results

### Anti-hGPA IgG Is Generated after RBC Transfusion in the Presence but Not the Absence of Poly IC

**Figure 1:**
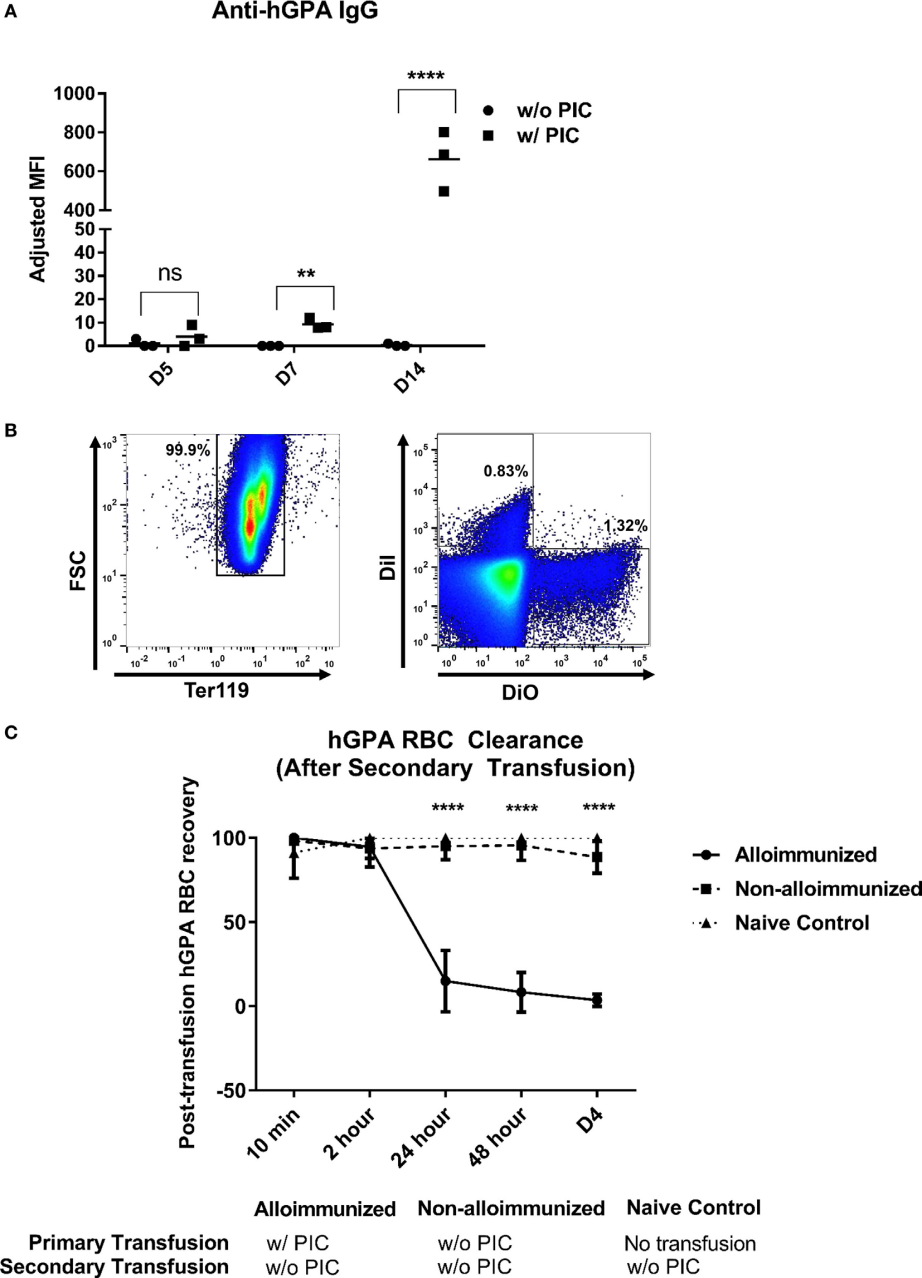
Characterization of immune responses to transfused human glycophorin A (hGPA) red blood cells (RBCs), in the presence or absence of poly IC. **(A)** Serum anti-hGPA IgG at day 5 (D5), day 7 (D7), and day 14 (D14) post-transfusion represented as adjusted mean fluorescence intensity (MFI) in mice transfused with hPGA RBCs in the presence or absence of pretreatment with poly IC. **(B)** Alloimmunized animals (previously transfused with hGPA RBCs in the presence of poly IC) or non-alloimmunized (previously transfused without poly IC) were transfused for a second time with DiI-labeled syngeneic FVB RBCs and 3,3′-dihexadecyloxacarbocyanine perchlorate (DiO) labeled hGPA RBCs; representative plot showing the gating strategy for DiO and DiO-positive RBCs (pregated on Ter119^+^ cells). **(C)** Post-transfusion clearance curve in alloimmunized, non-alloimmunized, or naïve mice. Data are representative of at least two experiments (*n* = 3–6 mice per group per experiment). **p < 0.01, ****p < 0.0001 determined by Mann–Whitney *U* test in panel **(A)** and ANOVA in panel **(C)**, between alloimmunized versus non-alloimmunized or naïve mice. There were no significant differences at any time point between naïve and non-alloimmunized mice.

Mice treated with poly IC approximately 4 h prior to hGPA RBC transfusion generated anti-hGPA IgG, whereas those transfused in the absence of poly IC did not form detectable anti-hGPA IgG at any evaluated time point (Figure 1A) (26).

We next sought to characterize GC formation and other immune cell responses in alloimmunized and non-alloimmunized animals. Splenic GC cells were not readily characterized in alloimmunized animals in this hGPA model by flow cytometry (Figure S1A in Supplementary Material). In parallel to the flow cytometric studies, we also imaged the spleen post-transfusion for well-defined GC areas. Consistent with the flow data, we found no statistically significant differences in the number of GCs between groups within the fields evaluated (Figure S1B in Supplementary Material). Additional studies were completed after repeat transfusion with no significant GC formation identified in alloimmunized animals (Figure S1C in Supplementary Material); antigen-specific B-cell evaluation completed using RBC membrane ghosts from hGPA donors also failed to show GC formation in this model (data not shown). Although it cannot be ruled out that anti-hGPA IgG is formed through a GC-independent pathway (34), our approach to detect GC B-cells (in the absence of being able to identify antigen-specific cells) in the hGPA system lacks sensitivity.

Along with GCs, other cells in the spleen and BM were characterized longitudinally after transfusion of hGPA RBCs in the presence or absence of poly IC treatment. The frequencies of total BM plasma cells (PCs) were not significantly different between mice transfused in the presence or absence of poly IC at any studied time point (Figure S2A in Supplementary Material). Foxp3^+^ Tregs as well as TFH cells (gated as CXCR5^hi^ PD1^+^ out of total TCRβ^+^ CD4^+^ T-cells) were evaluated at multiple time points (days 2–14) post-transfusion in the presence or absence of poly IC pretreatment; no differences in total cell numbers were observed between the groups at any studied time point (Figures S2B,C in Supplementary Material). Finally, CD5^+^ B-cells were evaluated, with no differences observed in gated cell numbers between any group studied (Figure S2D in Supplementary Material).

### Clearance of Transfused hGPA RBCs Occurs Only in Animals That Generate Anti-hGPA IgG

Antibodies of the IgG class that are specific for glycophorin may be clinically significant in humans, and we hypothesized that the anti-hGPA IgG generated by animals treated with poly IC prior to RBC transfusion would also be capable of leading to premature RBC clearance in our murine model. To investigate RBC clearance patterns, we labeled hGPA RBCs with one lipophilic dye and labeled wild-type FVB RBCs with a different lipophilic dye; a mixture of RBCs was then transfused in alloimmunized or non-alloimmunized animals and the ratio of circulating labeled FVB to hGPA RBCs was evaluated longitudinally post-transfusion (Figure 1B). In alloimmunized mice, there was rapid preferential clearance of 70–90% of transfused hGPA RBCs within the first 24 h, with almost all hGPA RBCs cleared by day 4 post-transfusion. In contrast, there was no preferential clearance of transfused hGPA RBCs in non-alloimmunized or naive mice by day 4 post-transfusion (Figure 1C) or beyond. These data highlight the clinical significance of the humoral immune response to hGPA RBCs in the presence of poly IC and also suggest the lack of “cellular immune”-mediated RBC clearance in animals that failed to generate an anti-hGPA IgG response after prior hGPA transfusion.

### Anti-hGPA IgG Formation Is Dependent on Recipient CD4 Cells

**Figure 2:**
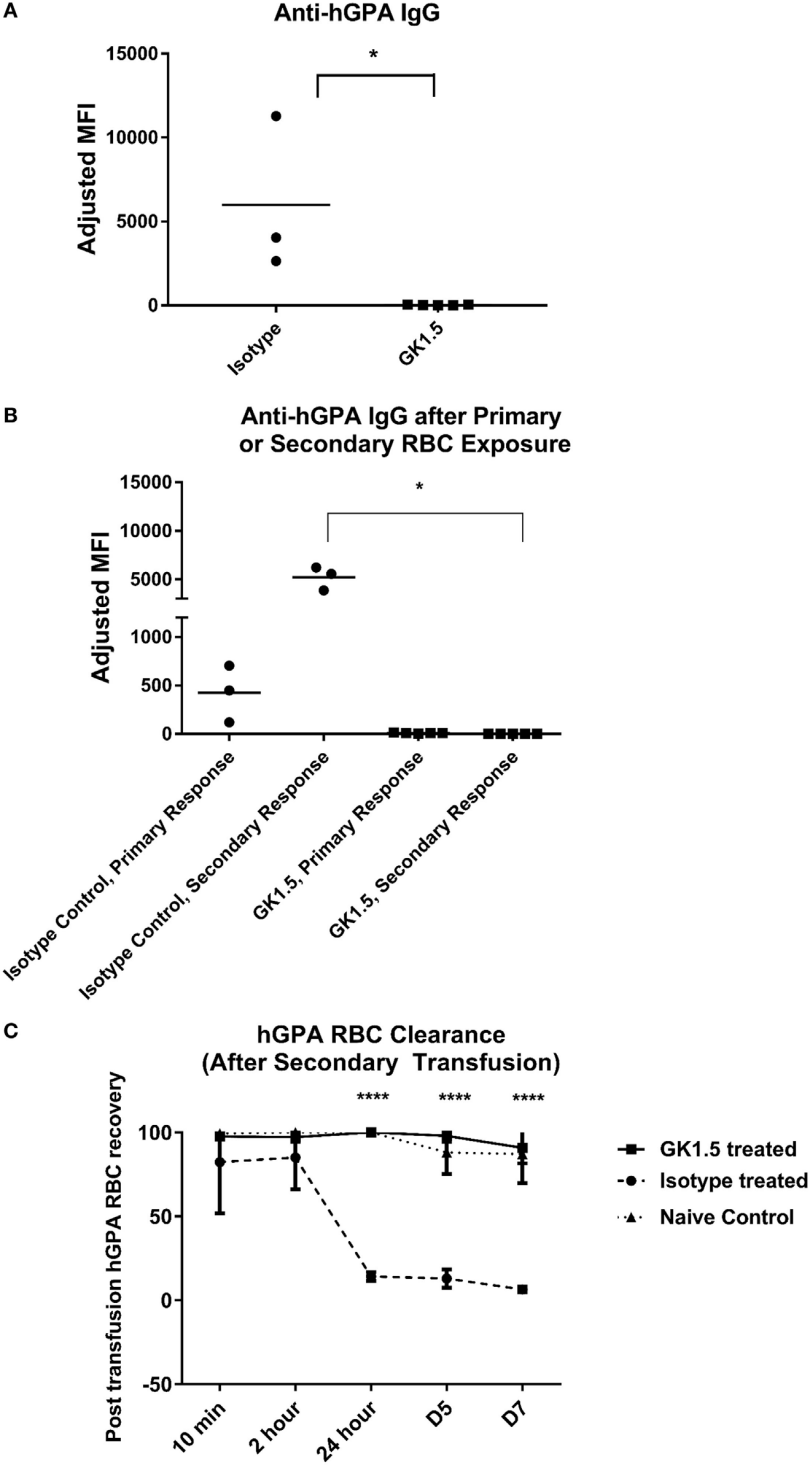
Alloantibody formation in the human glycophorin A (hGPA) RBC system is CD4 dependent. **(A)** Serum anti-hGPA IgG at day 14 represented as adjusted mean fluorescence intensity (MFI) in mice treated with GK1.5 or isotype-matched control antibody during primary transfusion. **(B)** Serum anti-hGPA IgG after primary or secondary hGPA RBC transfusion, with secondary transfusion given 5 weeks after primary transfusion and 3 weeks after last GK1.5 treatment. **(C)** Post-transfusion RBC clearance curve in GK1.5-treated, isotype-matched control antibody-treated, or naïve mice after secondary transfusion. *p < 0.05 determined by Mann–Whitney *U* test. ****p < 0.0001 determined by ANOVA between isotype control or naive and GK1.5-treated mice. There were no significant differences at any time point between naïve and isotype control mice. Data are representative of at least two experiments (*n* = 3 to 6 mice per group per experiment).

To determine whether the formation of RBC alloantibodies in the hGPA system was dependent on CD4 cells, we treated mice with CD4-depleting monoclonal antibody GK1.5 or an isotype-matched control antibody prior to and during primary transfusion with hGPA RBCs and poly IC (Figure S3 in Supplementary Material shows CD4 status at the time of transfusion). Anti-hGPA IgG was detectable only in isotype-matched control treated and not CD4-depleted animals on day 14 post-transfusion (Figure 2A).

Next, we sought to investigate the response to a secondary hGPA RBC challenge in mice previously transfused in a CD4-depleted status. To do this, mice previously treated with GK1.5 or an isotype control antibody during the primary transfusion were rested for 3 weeks; the CD4 status at the time of primary and re-transfusion is shown in Figure S3 in Supplementary Material. The animals were then treated with poly IC and re-challenged with hGPA RBCs. Despite having CD4 T-cells present during this second hGPA RBC transfusion, mice that saw the hGPA antigen initially in a state of CD4 depletion remained non-responders to the secondary RBC exposure (Figure 2B). In contrast, the isotype control antibody-treated mice had a significant boostable anti-hGPA IgG response to the secondary transfusion.

Finally, we investigated the fate of transfused hGPA RBCs in these animals. There was essentially no preferential hGPA RBC clearance over co-transfused antigen-negative FVB RBCs in animals treated with GK1.5 prior to their initial but not secondary RBC transfusion. In contrast, approximately 90% of transfused hGPA RBCs cleared in the control group within the first 24 h post-transfusion, with almost all hGPA RBCs being cleared within 5 days post-transfusion (Figure 2C).

Taken together, these data suggest that poly IC-driven hGPA alloimmunization is CD4 dependent, that conditions around the initial hGPA RBC exposure dictate secondary responses (e.g., CD4 depletion at primary RBC exposure induces tolerance to secondary exposures), and that only animals with detectable anti-hGPA IgG have premature clearance of incompatible transfused RBCs.

### Blocking CD40L Signaling Abrogates the Formation of hGPA Alloantibodies

**Figure 3:**
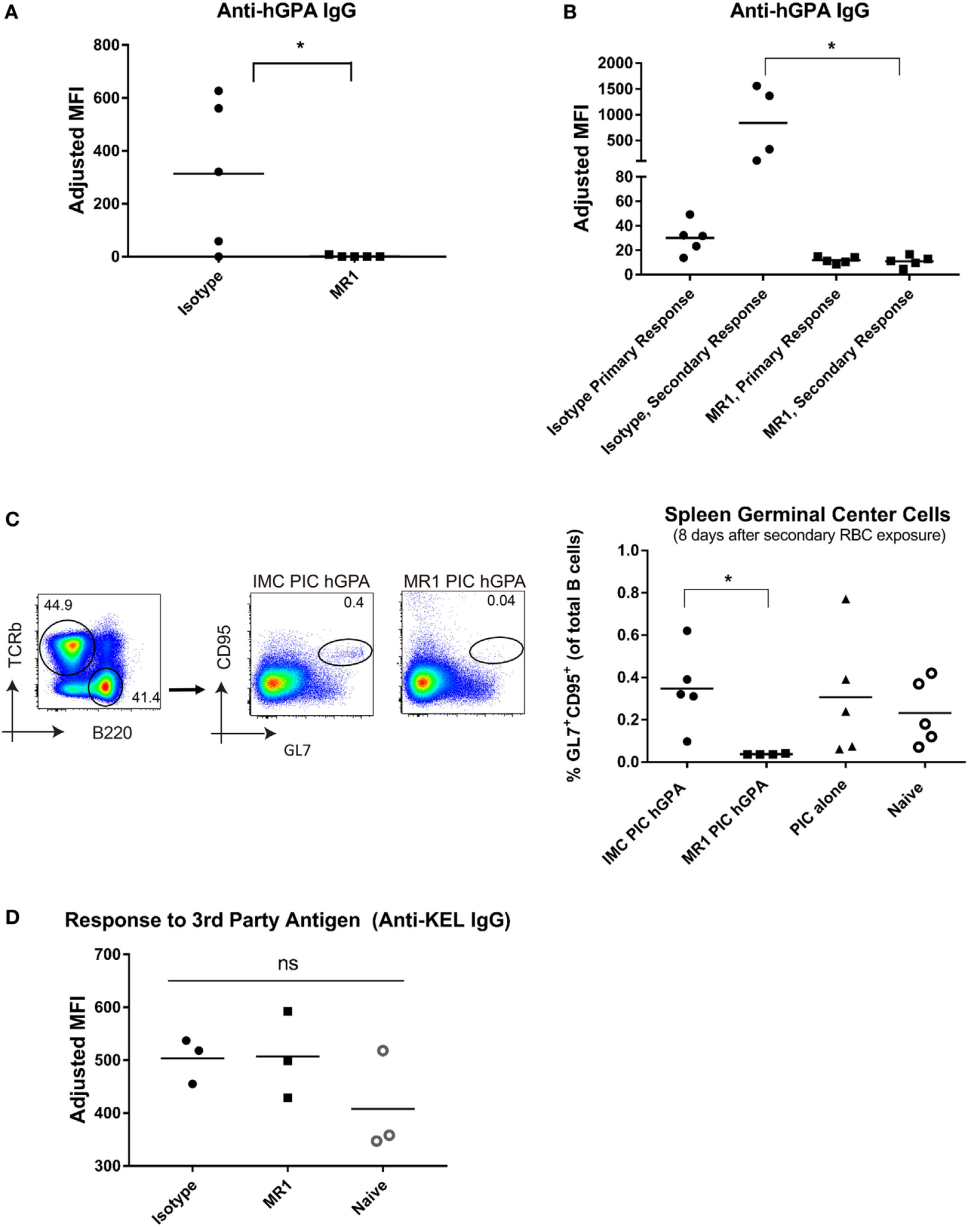
Alloimmunization to human glycophorin A (hGPA) RBCs is abrogated upon blocking CD40L, a co-stimulatory molecule. **(A)** Serum anti-hGPA IgG at day 14 post-transfusion represented as adjusted mean fluorescence intensity (MFI) in mice treated with MR1 or isotype antibody during primary transfusion. **(B)** Serum anti-hGPA IgG after primary transfusion (7 days before secondary transfusion) and 14 days after secondary transfusion. **(C)** Splenic germinal centers (GL7^+^ CD95^+^ B-cells) 8 days after secondary RBC exposure. **(D)** Serum anti-KEL IgG after primary KEL transfusion in naïve animals or in those previously treated with MR1 or isotype-matched control antibody during prior hGPA transfusion. *p < 0.05 determined by Mann–Whitney *U* test or ANOVA. Data are representative of at least two experiments (*n* = 3–5 mice per group per experiment).

Since CD40-CD40L signaling is critical in both antigen-presenting cell (APC):CD4 interactions (35) and T-cell:B-cell interactions (36, 37), we sought to determine if blocking this signaling could prevent hGPA RBC alloantibody formation. Mice were treated with the anti-CD40L monoclonal antibody, MR1, or an isotype-matched control, prior to treatment with poly IC and transfusion with hGPA RBCs. MR1-treated mice failed to generate detectable anti-hGPA IgG (Figure 3A shows data from 14 days post-transfusion). As we observed with CD4-depleted animals, mice initially transfused in the presence of CD40L blockade failed to generate detectable anti-hGPA IgG when treated with poly IC and re-transfused with hGPA 5 weeks later (Figure 3B). In contrast, the isotype control antibody-treated mice demonstrated a boostable anti-hGPA IgG response. These data suggest that the transfusion recipient’s CD40/CD40L interaction at first hGPA RBC exposure dictates subsequent hGPA responses.

Next, splenic GC responses were evaluated in animals 8 days following their secondary RBC exposure. Animals transfused in the presence of MR1 had essentially no detectable GL7^+^ CD95^+^ B-cells (Figure 3C). In contrast, those transfused in the presence of an isotype type-matched controls had GCs (albeit no more than those found in naïve animals or in animals treated with poly IC alone).

### Non-Responsiveness to Subsequent hGPA RBC Exposure after CD40L Blockade Is Antigen Specific

To investigate whether CD40L blockade during initial hGPA RBC exposure led to diffuse RBC non-responsiveness or to antigen-specific RBC non-responsiveness, we exposed alloimmunized and non-alloimmunized mice to a third-party antigen (KEL glycoprotein) on transfused RBCs. The immune response induced by the KEL-expressing RBCs used for these experiments (KEL^hi^ RBCs) is T-cell dependent (data not shown, manuscript in preparation). Serum was collected longitudinally post-transfusion, with flow cytometric crossmatch assays completed using transgenic KEL or wild-type RBCs as targets. MR1-treated mice, isotype control mice, and naïve WT mice had similar peak levels of anti-KEL antibodies (Figure 3D), suggesting antigen-specific non-responsiveness.

### CD40L Blockade Prevents Expansion of Antigen-Specific CD4^+^ T-Cells, Prevents GC Formation, and Blunts HOD RBC Alloantibody Responses

To investigate the potential mechanisms of action of CD40L blockade using MR1 Ab in a transfusion setting, we turned to a transgenic murine model that enables the study of antigen-specific CD4^+^ T-cells. HOD transgenic mice have RBC-specific expression of the hen egg lysozyme, ovalbumin, and the human Duffy antigen, and OTII CD4^+^ T-cells have been shown to proliferate in recipients transfused with HOD RBCs (12, 38). For these experiments, naïve C57BL/6 mice were adoptively transferred with 10,000 CD45.1^+^ OTII CD4^+^ T-cells and then transfused with HOD RBCs and poly IC with or without MR1 treatment. MR1-treated mice demonstrated significant blunting of peak anti-HOD IgG responses compared to those transfused in the absence of MR1 treatment (Figure S4 in Supplementary Material).

**Figure 4:**
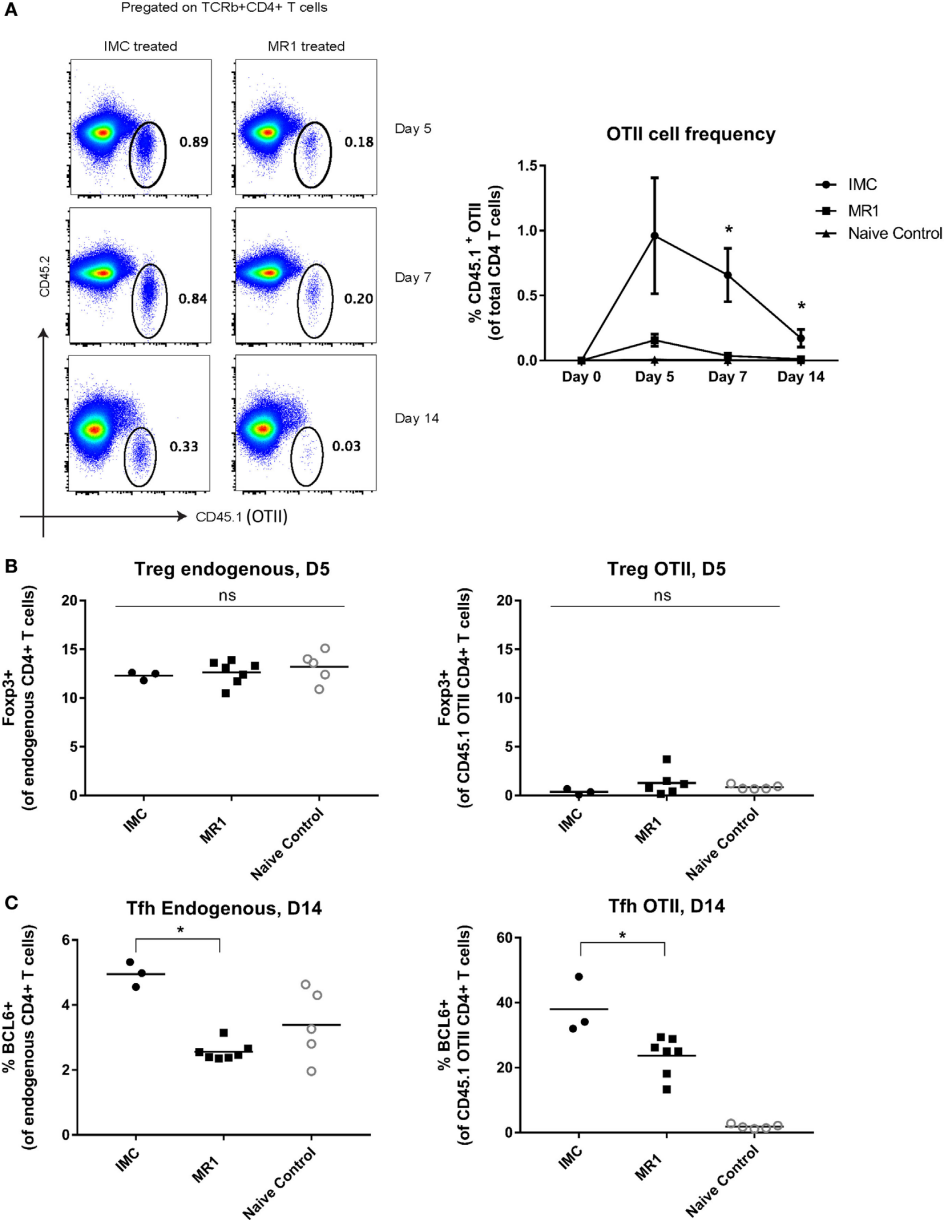
CD40/CD40L blockade prevents expansion of antigen-specific CD4^+^ T-cells. **(A)** Representative flow plots showing gating strategy for OTII cells gated as CD45.1^+^ CD4^+^ T-cells, and frequency of OTII cells at days 0, 5, 7, and 14 post-transfusion in mice treated with or without MR1. **(B)** Endogenous and OTII regulatory T-cells (Tregs) (Foxp3^+^ CD4^+^ T-cells). **(C)** Endogenous and OTII TFH (BCL6^+^). *p < 0.05 determined by Mann–Whitney *U* test or ANOVA. Data are representative of at least two experiments (*n* = 3–6 mice per group per experiment).

To determine the effect of MR1 treatment on the expansion of antigen-specific T-cells, we evaluated the frequency of antigen-specific CD45.1^+^ OTII cells in transfusion recipients at multiple time points post-transfusion (Figure S4 in Supplementary Material and Figure 4A show representative gating). The frequency of OTII cells was significantly lower in the MR1-treated group compared to the isotype-matched control treated group on days 7 and 14 post-HOD transfusion (Figure 4A). Given prior studies of MR1 and Tregs in graft-versus-host disease (GVHD) models (39), we evaluated these cells in our transfused animals. We found no significant differences in the number of endogenous or antigen-specific (CD45.1^+^ OTII) Foxp3^+^ CD4^+^ Tregs between groups (Figure 4B), with there being extremely few antigen-specific OTII cells to characterize in animals treated with MR1. To further investigate whether Tregs may play a critical role in the mechanism of action of MR1 alloantibody mitigation, we depleted Tregs using PC61 antibody (BioXcell), treated animals with MR1, transfused with transgenic RBCs, and evaluated alloantibody response 2 weeks later. MR1 treatment prevented RBC alloimmunization in both Treg-depleted and Treg-replete mice in this pilot experiment (data not shown), suggesting that Treg expansion is not necessary for the mechanism of action of MR1 in this system. We also evaluated endogenous and antigen-specific TFH cells in the transfused animals and found significantly decreased TFH cell numbers in animals treated with MR1 compared to those treated with the isotype-matched control (Figure 4C).

**Figure 5:**
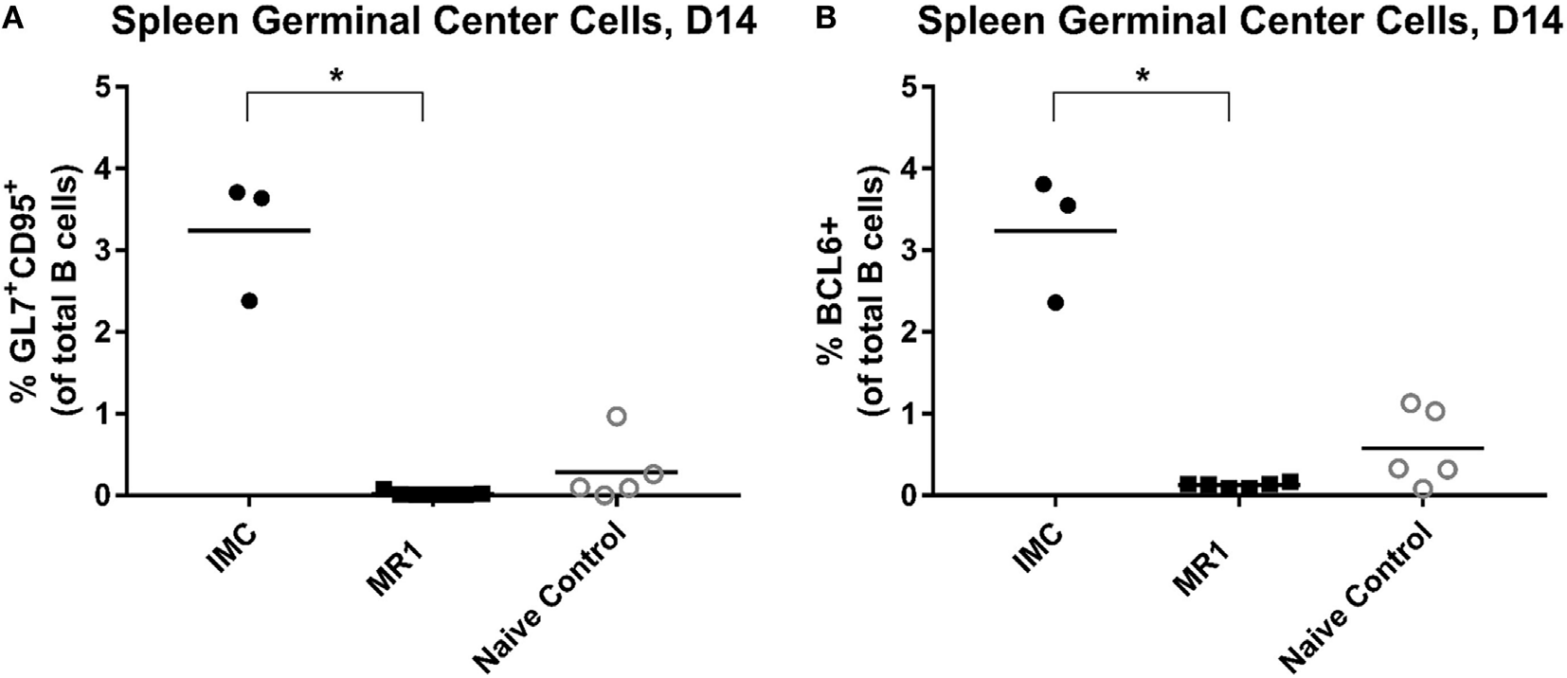
CD40/CD40L blockade prevents germinal center formation after HOD (hen egg lysozyme, ovalbumin, and human duffy^b^) transfusion. **(A)** Splenic germinal centers (GL7^+^ CD95^+^ B-cells) or **(B)** BCL6^+^ B-cells cells, 14 days after primary HOD RBC exposure. *p < 0.05 by ANOVA. Data are representative of two experiments (*n* = 3–6 mice per group per experiment).

Finally, we evaluated splenic GC responses in these transfusion recipients. HOD RBCs were able to induce robust GC formation following a single transfusion. Treatment with MR1, however, fully prevented GC formation in transfused animals (Figures 5A,B).

## Discussion

In this article, we have shown the importance of recipient CD4^+^ cells and CD40L in the alloimmune response to transfused murine RBCs expressing the hGPA antigen. Poly IC or some adjuvant is required in this model to lead to an alloimmune response, which is consistent with data emerging in humans about a correlation between certain types of recipient inflammation and alloimmunization (7, 40). Notably, any initial hGPA RBC exposure that resulted in a non-detectable anti-hGPA humoral response appeared to tolerize the transfusion recipient in an antigen-specific manner, with future hGPA but not third-party antigen RBC exposures failing to induce detectable alloimmune responses.

The fact that an adjuvant is required in this model for a humoral immune response to occur, in combination with the fact that CD4 depletion or CD40L blockade can induce complete and long-lasting immune tolerance in an antigen-specific manner, suggest but do not conclusively confirm that immune responses to antigens on transfused RBCs are distinct from those to other antigens administered in a soluble form or through different routes. For example, although CD40L blockade has been shown to decrease skin and organ transplant rejection in mice and in non-human primates, suppression of the recipient CD8^+^ T-cell component of transplant rejection may require additional co-stimulatory molecule blockade or immunosuppression (39, 41, 42). Further, although CD40L blockade has been shown to prevent a primary immune response to factor VIII exposure in murine models of hemophilia (43), this non-responsiveness does not equate to long-lasting tolerance. By virtue of their circulation solely in the intravascular space and their sterile nature, RBCs may in fact be unique vehicles through which to induce tolerance (44). To date, there have been no studies in humans to determine whether non-responsiveness to RBC antigens (defined as the lack of a detectable anti-RBC alloantibody response after a transfusion) equates to tolerance or is simply due to ignorance. Data documenting low rates of RBC alloimmunization in chronically transfused patients with hemoglobinopathies initially exposed to RBCs at young ages (45–47) lend support to the idea of tolerance induction. Recent studies by Hubbell et al., comparing responses of soluble antigen to that of the same antigen associated with RBCs, demonstrate differences in the way the immune system views and responds to the same antigen presented in a different manner (48–50). These cited studies, undertaken with the hypothesis that RBC-surface bound antigen would be cleared tolerogenically along with eryptotic debris, also highlight the role of recipient T-cells in the tolerance induction process (49).

Our studies build upon the past work of responders/non-responders to transfused RBC antigens published by others (25, 51, 52). Given the lack of antigen-specific tools for studies in the hGPA system, we turned to the HOD model for mechanistic investigation. Our data using CD40L blockade in the HOD system demonstrated the prevention of antigen-specific CD4^+^ T-cell expansion. Although an increased ratio of Tregs to Teff has been observed with CD40L blockade in murine GVHD models (39, 53), our data suggest that Tregs do not appear to be critically important to the prevention of HOD RBC alloimmunization by MR1 treatment.

In addition to impacting Treg/Teff ratios, studies in other models suggest that CD40L blockade also impacts APC cytokine production and/or downstream B-cell responses. This blockade has been shown to be important not only for cytokine production from APCs (31) but also for upregulation of ICAM-1, CD80, and CD86 on these cells (54). One cytokine of particular interest in RBC alloantibody responses is IL-6, with a recent manuscript describing the critical importance of IL-6 and IL-6Rα signaling on CD4^+^ T-cells in the development of antibodies against HOD RBCs (38). Of further interest is the role that bridging channel dendritic cells play in immune responses to transfused RBCs, given the recently described role of 33D1 CD4^+^ conventional dendritic cells in mediating alloantibody responses to HOD RBCs (12). The CD40L signaling axis has been shown to be required for B-cell proliferation and survival and for sustaining TFH cells during GC responses (55). Consistent with this, we observed that MR1-treated animals had no GC formation in studies in the hGPA and HOD systems. Future studies investigating CD4^+^ T-cell/B-cell interactions are warranted, with one unique aspect of antigens on RBCs being their ability to crosslink B-cell receptors. Studies completed in a different murine model have shown that the tolerance induced following transfusion of RBCs expressing the KEL glycoprotein at very low density (KEL^lo^ RBCs) can be adoptively transferred *via* B-cells from non-responder animals to naïve, irradiated, MuMT mice (56). These data highlight the potential importance of B-cells in RBC antigen-induced tolerance induction and maintenance.

Our studies focused on humoral alloimmunity, as alloantibodies are a measurable and clinically relevant immunologic outcome in transfusion medicine. However, it has recently been appreciated that recipient CD8^+^ T-cells may play a role in the antibody-independent premature clearance of transfused platelets in a murine model (57). In our current study, we thus compared the clearance of transfused hGPA RBCs in non-alloimmunized and alloimmunized animals, as a first evaluation of whether cellular mediated immunity may have been induced in animals who lacked a detectable humoral immune response. No premature hGPA RBC clearance was observed after transfusion in any non-alloimmunized animal lacking detectable anti-hGPA alloantibodies. Despite this lack of an obvious “cellular immune response,” it cannot be ruled out that transfusions even under “tolerogenic” conditions may have additional immunologic sequelae. For example, it is at least theoretically possible that transfusions may be able to induce cell-mediated immunity capable of priming a recipient for hematopoietic stem cell transplant rejection, with such rejection previously described in murine models involving platelet or RBC transfusions (28, 58).

We completed the described studies in the hGPA system, given the clinical relevance of this human blood group and the responder/non-responder status we observe in recipients transfused in the presence or absence of an adjuvant. However, the inability to evaluate hGPA antigen-specific T-cells or B-cells limits mechanistic study options in this system and also limits the value of descriptive characterizations of GCs, PCs, and the like. Another limitation includes the inability to study hGPA responses in animals genetically lacking cell subsets or pathways, as few such animals exist on the same genetic (FVB/H2-q) background. In this article, we describe non-responder mice as being tolerized, yet future studies in other model systems are needed to fully characterize which recipient cell subsets may be able to transfer tolerance to naïve recipients. We focused our co-stimulatory molecule studies on CD40L blockade, although this is but one of many co-stimulatory/co-inhibitory pathways to investigate. As limitations to the translation of any CD40L blockade therapy may involve the risk of thromboembolism due to CD154 expression on platelets (59), blocking therapies that do not directly impact CD40L are actively being investigated in other models (60).

In conclusion, our data show the importance of recipient CD4^+^ cells and the CD40/CD40L interaction (potentially between APC:T-cells or T-cells:B-cells) in determining whether a recipient becomes alloimmunized or tolerized to antigens on transfused RBCs. Our studies lay the groundwork for future studies investigating responsiveness/non-responsiveness to transfused RBC antigens in animal models and in humans and for expanding studies evaluating RBCs as vehicles through which to induce tolerance.

## Ethics Statement

All procedures and protocols were approved by Emory and Yale University’s Institutional Animal Care and Use Committee.

## Author Contributions

PN, DL, SP, SS, and JH designed the experiments and performed the primary experiments; JL, MS, DG, and DM assisted with the experiments; PN, DL, and JH analyzed the results and made the figures; PN and JH wrote the manuscript; and all authors edited the manuscript.

## Conflict of Interest Statement

The authors declare that the research was conducted in the absence of any commercial or financial relationships that could be construed as a potential conflict of interest.
