# Supplementary material for: CD4 Depletion or CD40L Blockade Results in Antigen-Specific Tolerance in a Red Blood Cell Alloimmunization Model
Source: Front Immunol. 2017 Aug 7;8:907. doi: 10.3389/fimmu.2017.00907 (PMC5545689; doi:10.3389/fimmu.2017.00907)

Supplementary Material

CD4 depletion or CD40L blockade results in antigen specific tolerance in an RBC alloimmunization model

Prabitha Natarajan, Dong Liu, Seema R. Patel, Manjula Santhanakrishnan, Daniel Beitler, Jingchun Liu, David R. Gibb, Justine S. Liepkalns, David J. Madrid, Stephanie C. Eisenbarth, Sean R. Stowell, and *Jeanne E. Hendrickson

***Correspondence:** Jeanne Hendrickson, email: jeanne.hendrickson@yale.edu

Supplementary Figure 1. Characterization of germinal center cells after transfused hGPA RBCs, with or without poly IC. (A) Representative flow plots showing gating strategy in splenocytes; B cells (pre-gated on lymphocytes) were gated as $CD19^+$ $B220^+$ and GC B cells were sub-gated as $GL7^+$ $CD95^+$; frequency of GC B cells in the spleens of mice 6, 8, and 12 days post transfusion with hGPA RBCs in the absence or presence of poly IC, compared to mice treated with poly IC alone or naïve mice. **(B)** Representative image of spleen day 8 post transfusion; IgD shown in red, GL7 in white, and CD4 in blue. The white clusters within a B cell follicle were counted as GCs; the number of GCs in the entire field of view of an 8µm section is shown. Data in A and B are representative of at least 2 experiments (n=3 to 6 mice per group per experiment); data in C is from a single experiment (n=5 mice per group).

Supplementary Figure 1

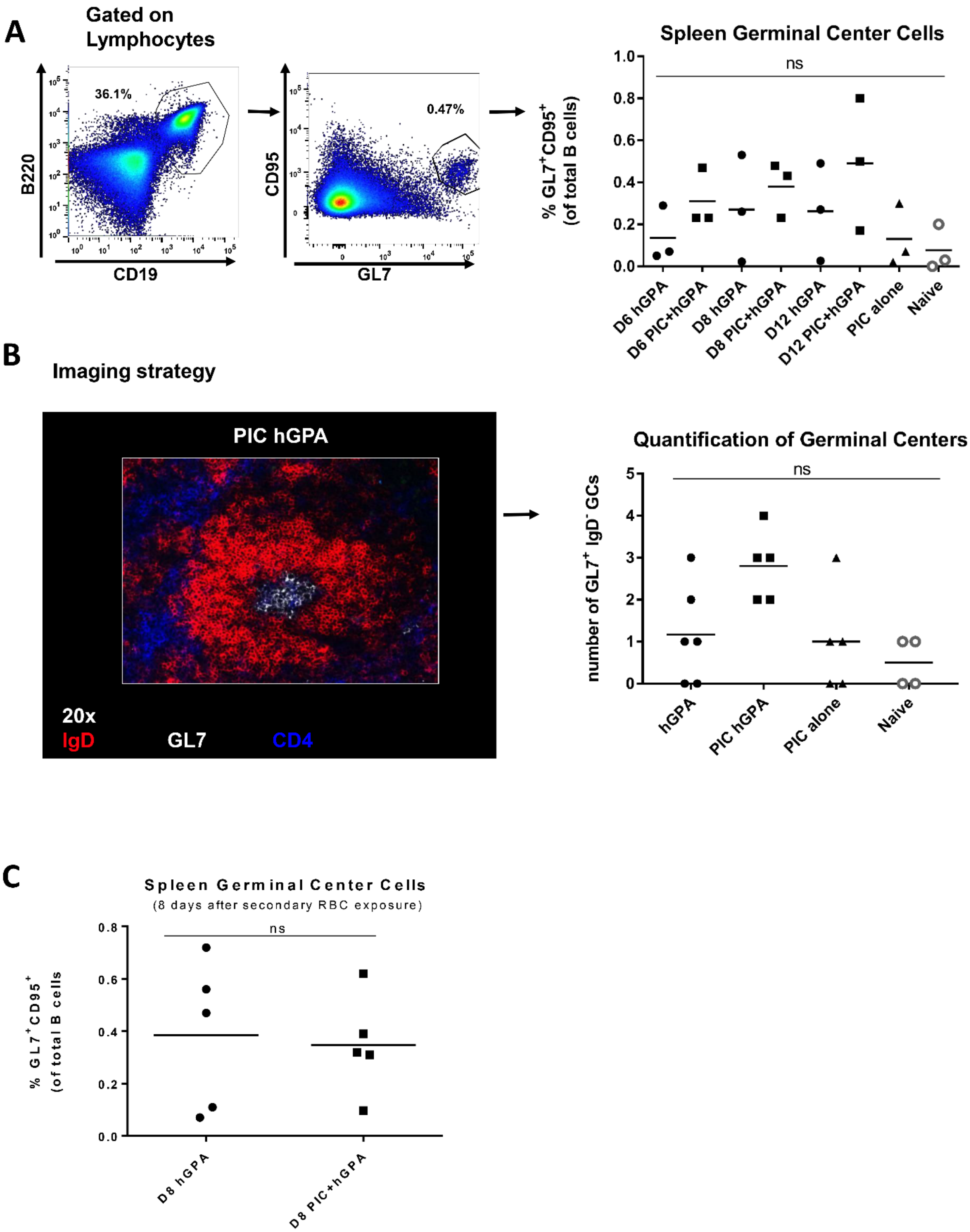

Supplementary Figure 2. Characterization of additional immune response to transfused hGPA RBCs, with or without poly IC. (A) Frequency of plasma cells (PCs, gated as $CD138^+$ $B220^{lo}$ cells) out of total lymphocytes in bone marrow of naïve mice or those 14 days after hGPA transfusion in the presence or absence of poly IC. **(B)** Frequency of Tregs (gated as $Foxp3^+$ cells) out of total CD4 T cells in spleens of naïve mice or of those 14 days after transfusion with hGPA RBCs in the presence or absence of poly IC. **(C)** Representative flow plots show gating strategy for T follicular helper T cells (Tfh cells); $CD4^+$ T cells were gated as $TCR\beta^+$ $CD4^+$, and Tfh cells were sub-gated as CXCR5hi $PD1^+$ cells; frequency of TFH cells out of total CD4 T cells in spleens of naïve mice or those 4 days after transfusion with hGPA RBCs in the presence or absence of poly IC. **(D)** Frequency of $CD5^+$ cells out of total B cells in spleens of naïve mice or of those 14 days after transfusion with or without PIC. Data are representative of at least 2 experiments (n=3 to 6 mice per group per experiment). Kruskal Wallis with Dunn's post-test was performed to determine significance.

Supplementary Figure 2

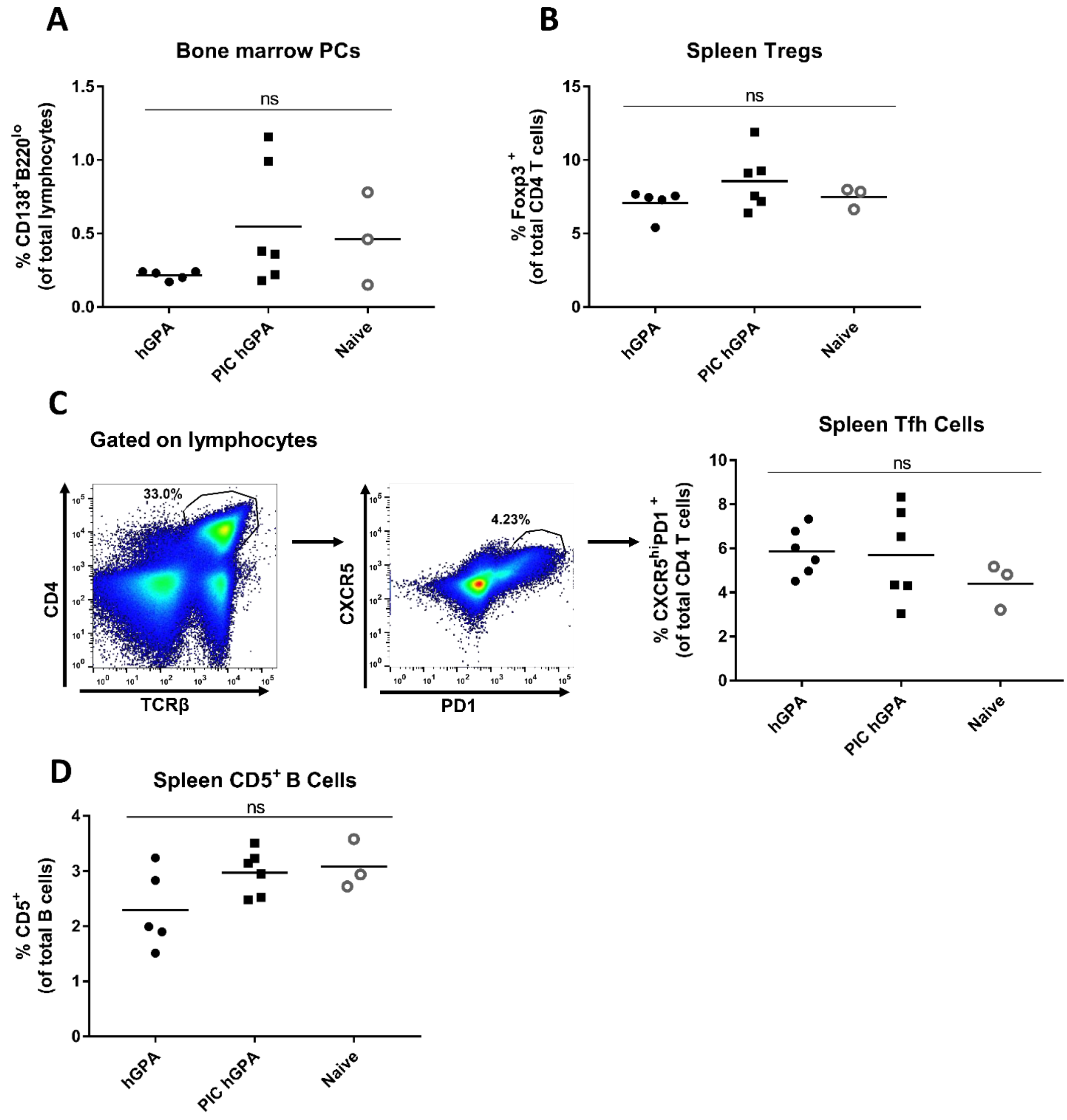

Supplementary Figure 3. Recipient CD4+ T-cell status after GK1.5 treatment and prior to secondary RBC exposure. (A) CD4+ T-cells immediately after GK1.5 treatment, at the time of initial RBC exposure. **(B)** CD4+ T-cells 3 weeks after GK1.5 treatment, at the time of secondary RBC exposure.

Supplementary Figure 3

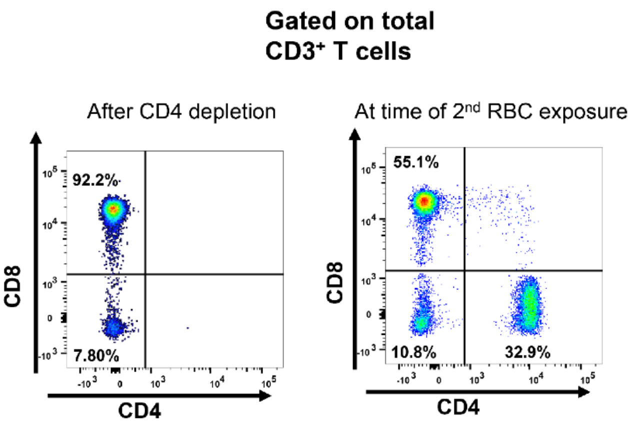

Supplementary Figure 4. Immune responses after OTII adoptive transfer and transgenic HOD RBC transfusion. (A) Serum anti-HOD IgG at day 14 post-transfusion, represented as adjusted mean fluorescence intensity (MFI) in mice transfused with HOD RBCs in the presence or absence of MR1. *p<0.05 determined by Mann Whitney U test. **(B)** Representative flow cytometric gating of adoptively transferred CD45.1+ OTII cells.

Supplementary Figure 4

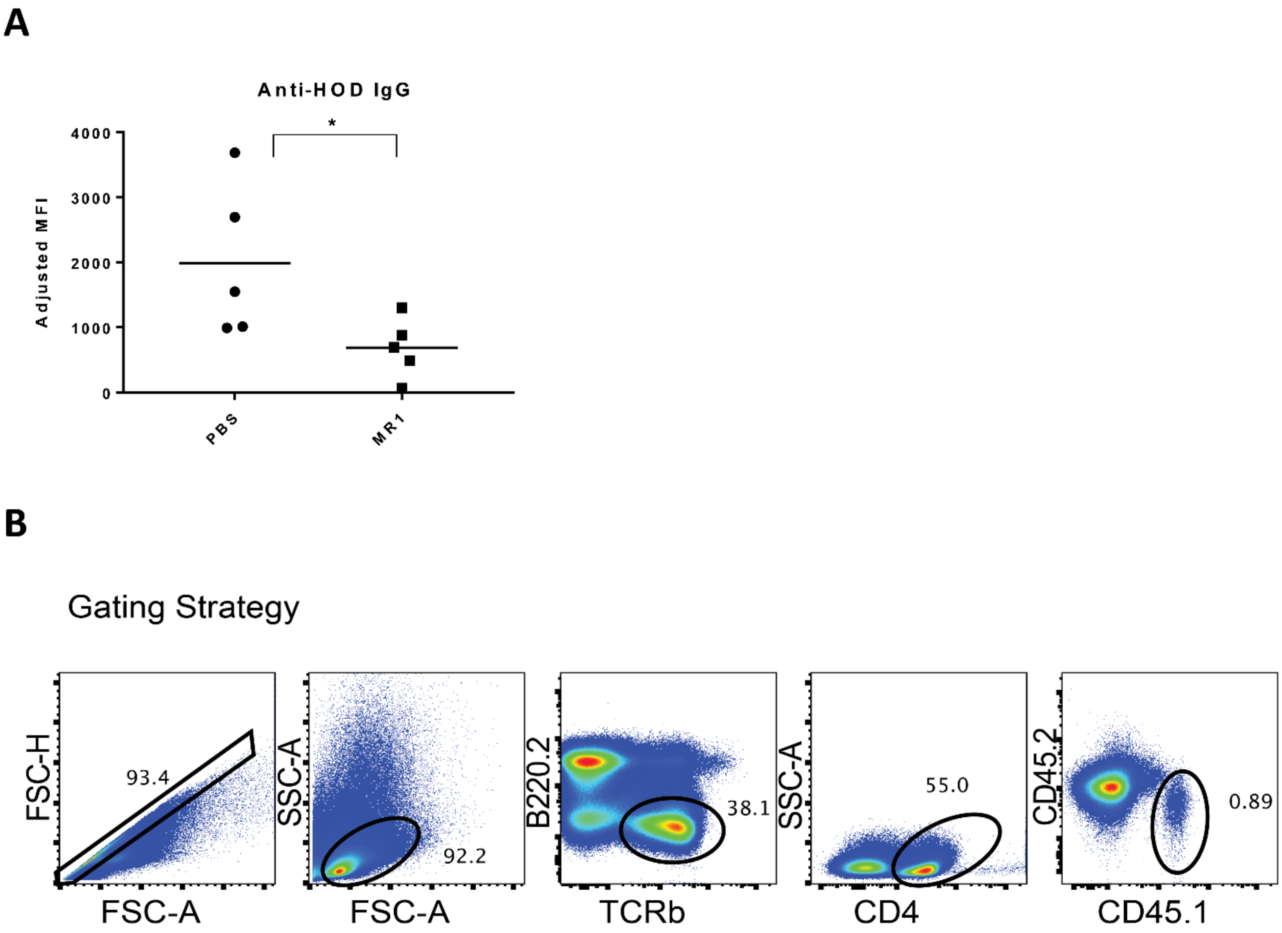

Supplement: Supplementary file 1 [file data_sheet_1.pdf]
